# Treatment of rigid post-traumatic thoracolumbar kyphosis by a novel technique of spinal joints release

**DOI:** 10.1186/s13018-023-03599-7

**Published:** 2023-02-21

**Authors:** Qing Wang, Chao Tang, GaoJu Wang, GuangZhou Li, DeJun Zhong, Song Wang, Fei Ma

**Affiliations:** 1 Department of Orthopaedics, The Affiliated Hospital of Southwest Medical University, No. 25 Taiping Street, Lu Zhou, Sichuan, 646000 China; 2 Department of Orthopedics, Xin Qiao Hospital, Army Medical University (Third Military Medical University), Chongqing, China

**Keywords:** Post-traumatic thoracolumbar kyphosis, Spinal joints release, Surgical technique, Feasibility, Clinical efficacy

## Abstract

**Objective:**

The purpose of this study was to evaluate the feasibility of a novel technique named spinal joints release (SJR) and observe its efficacy in treating rigid post-traumatic thoracolumbar kyphosis (RPTK).

**Methods:**

RPTK patients who were treated by SJR with facet resection, limited laminotomy, clearance of the intervertebral space, and release of the anterior longitudinal ligament through the intervertebral foramen and disc of injury segment from August 2015 to August 2021 were reviewed. Intervertebral space release, internal fixation segment, operation time, and intraoperative blood loss were recorded. The intraoperative, postoperative, and final follow-up complications were observed. An improvement in the VAS score and ODI index was observed. Spinal cord functional recovery was evaluated by American Spinal Injury Association Impairment Scale (AIS). Improvement of local kyphosis (Cobb angle) was evaluated by radiography.

**Results:**

Forty-three patients were successfully treated by the SJR surgical technique. Open-wedge anterior intervertebral disc space was performed in 31 cases, and repeated release and dissection of the anterior longitudinal ligament and callus were performed in 12 cases. There was no lateral annulus fibrosis release in 11 cases, the anterior half release of lateral annulus fibrosis in 27 cases, and complete release in five cases. There were five cases of screw placement failure in one or two side pedicles of the injured vertebrae due to excessive resection of the facets and improper pre-bending of the rod. Sagittal displacement occurred in four cases at the released segment due to the complete release of bilateral lateral annulus fibrosus. Autologous granular bone + Cage was implanted in 32 cases, and autologous granular bone was implanted in 11 cases. There were no serious complications. The average operation time was 224 ± 31 min, and intraoperative blood loss was 450 ± 225 mL. All the patients were followed up with an average of 26 ± 8.5 months. The VAS scores and ODI index improved significantly at the final follow-up. All of the 17 patients with incomplete spinal cord injury achieved more than one grade of neurological recovery at the final follow-up. An 87% correction rate of kyphosis was achieved and maintained, with the Cobb angle decreasing from 27.7° preoperatively to 5.4° at the final follow-up.

**Conclusion:**

Posterior SJR surgery for patients with RPTK has the advantages of less trauma and less blood loss, and kyphosis correction is satisfactory.

## Introduction

It has been reported that rigid post-traumatic thoracolumbar kyphosis (RPTK) greater than 20° and combined with severe pain or progressive neurologic deficit requires surgical correction [1]. Common orthopedic strategies for RPTK include anterior subtotal vertebral resection, posterior Smith–Petersen osteotomy (SPO), pedicle subtraction osteotomy (PSO), and posterior–anterior approach [1–13]. Many surgeons have used grade III or grade IV osteotomy techniques for RPTK [6–9] since Schwab proposed a systematic osteotomy technique for kyphosis in 2014 [5]. However, the surgical results are not always satisfactory because the potentially damaged intervertebral disc is preserved, and all the posterior elements are resected during this procedure. In addition, there are some complications for these osteotomy techniques such as a large amount of osteotomy, severe trauma, excessive blood loss, severe spinal cord shortening, and spinal cord injury.

According to previous literature and clinical observations, the main imaging features of RPTK patients are as follows: (1) severe wedge-shaped lesions of the injured vertebra [14]; (2) scar formation of the anterior longitudinal ligament, and callus can be found in some patients [15–17]; (3) posterior facet stiffness with spontaneous traumatic bone fusion [18]; (4) a certain distance between the anterior longitudinal ligament callus and the abdominal aorta and vein. Therefore, we developed a novel technique of spinal joints release (SJR) in which the intervertebral soft tissue and callus should be cleared and released from posterior to anterior through the foramina and injured disc. This technique avoids extensive osteotomy and injury of perivertebral blood vessels, restores vertebral height as to fracture reduction, and avoids spinal cord shortening. With this new technique, the spinal cord sequence can be restored during the correction of thoracolumbar kyphosis.

The purpose of this study was to report the radiographic and clinical results of SJR for the correction of RPTK and evaluate the feasibility of the novel technique in treating patients with RPTK.

## Materials and methods

### Samples

The study was approved by the Ethics Committee of the Affiliated Hospital of Southwest Medical University. RPTK patients who had been treated with our self-designed SJR technology from August 2015 to August 2021 were reviewed. Inclusion criteria were as follows: (1) history of trauma, old fracture from T11 to L2 with kyphosis (Cobb angle more than 20°), and local segmental stiffness on dynamic radiography; (2) lower back pain with or without bilateral lower limb pain, numbness, weakness, or urination problems for more than three months, where conservative treatment was invalid; (3) complete preoperative and postoperative clinical and imaging data available. Exclusion criteria were as follows: (1) combination with other spinal fractures; (2) ankylosing spondylitis, old tuberculosis, and kyphosis caused by osteoporotic vertebral compression fracture; (3) brain, cervical spinal cord, or thoracic spinal cord injury and inflammation; (4) mental disease and the inability of a patient to cooperate.

There were 43 cases, including 20 males and 23 females. The average age was 52.8 ± 11 years (23–71 years). The mean time from trauma to surgery was 5.7 ± 8.9 years and ranged from 3 months to 40 years. There were three cases of RPTK, one case of posterior Harrington rod fixation, and two cases with kyphosis after the removal of posterior internal fixation. The general clinical information of the patients is detailed in Table 1. Old fracture of T11, T12, L1, and L2 was involved in 4, 11, 20, and 8 cases. Compression fracture occurred in 21 cases, and burst fracture was found in 22 cases. Anterior callus or bone bridge formation of the injured spine was seen in 13 cases (30%). In 16 patients (37%), discs intruded into the injured vertebrae (in 11 cases through the upper-end plate, in three cases through the lower-end plate, and in two cases through the upper and lower-end plates). Traumatic or spontaneous fusion of posterior facet joints was found in 31 cases (72%), including 27 cases at the single level and four cases at the double level. The American Spinal Injury Association Impairment Scale (AIS) was used to classify the severity of spinal cord injury. There were 17 cases of incomplete spinal cord injury (grade C of AIS in four cases, grade D of AIS in 13 cases). The local Cobb angle of the thoracolumbar segment was 27.7° ± 7.8° (18°–49°).

**Table 1:** Data of the 43 patients with rigid post-traumatic kyphosis

NO	Sex	Age (yrs)	During (yrs)	Level	Type	Callus	Collapse^*^	Facet fusion^#^	AIS grade	Cobb angle (°)			Complication		Follow up (yrs)	BMD
										Pre-O	Post-O	Fu	Operation	Post-O		
1	M	55	34	L1	C	No	No	Yes	C	26	-4	3	X3	No	1.5	− 1.8
2	M	54	3	T12	C	No	Yes	No	E	23	8.7	9.5	No	No	5.3	− 1.5
3	F	34	10	L1	B	No	Yes	Yes	E	28	4	6	No	CSF	0.6	–
4	M	62	20	L1	B	No	No	Yes	D	31	11	12	X1	No	1.5	− 1.5
5	M	40	0.4	T12	C	No	No	No	E	22	5	7	No	No	3.5	–
6	F	53	0.3	L1	B	No	No	No	D	22	1	3	No	IN	2.1	− 2.0
7	M	65	4	L1	B	Yes	Yes	Yes	C	40	9	12	X3, X4	CSF	2.5	− 2.2
8	F	49	0.6	L1	B	No	No	No	E	21	-4	0	No	No	2.1	–
9	F	54	0.4	L1	C	No	No	No	E	23	3	5	X3	No	2.5	− 1.8
10	M	50	12	T11	C	No	Yes	Yes	E	22	4	5	X1, X2	CSF	3.5	− 0.5
11	M	68	1.1	L1	C	No	No	Yes	E	22	6	8	No	IN	0.5	− 1.5
12	M	64	40	L1	B	Yes	Yes	Yes	D	49	3	3	X1, X2, X3	No	3	− 2.3
13	F	71	0.4	T12	B	No	Yes	Yes	E	31	7	9	No	DVT	2.5	− 2.5
14	M	70	0.7	L2	B	Yes	Yes	Yes	D	22	0	3	No	No	2.5	− 1.0
15	M	56	0.3	L2	B	Yes	No	Yes	D	20	1	3	No	No	2.4	− 1.5
16	F	51	0.9	L1	C	No	No	Yes	D	24	-3	0	No	No	3.0	− 2.0
17	F	68	0.3	T12	B	No	No	No	C	23	6	8	No	CSF	2.6	− 2.5
18	F	57	10	T12	C	No	No	Yes	E	30	7	9	No	No	1.4	− 2.4
19	F	54	12	L1	B	Yes	Yes	Yes	E	24	5	8	No	No	1.5	− 1.7
20	F	51	6	L2	B	Yes	No	Yes	E	18	-2	2	No	No	2.1	− 2.0
21	F	54	0.3	T12	B	No	No	No	E	25	3	5	No	No	1.4	-2.0
22	F	47	0.3	L1	B	No	Yes	Yes	E	22	0	5	No	No	0.5	–
23	M	44	0.7	L1	C	No	No	Yes	D	25	8.3	8.5	No	No	3.5	–
24	M	66	2.1	L2	C	No	No	Yes	E	32	5	7	No	No	3.2	− 2.0
25	F	32	7	L1	C	Yes	Yes	Yes	D	45	7	9	X4	IN	1	–
26	M	45	1	L1	B	No	No	No	E	23	3	3	No	No	2	–
27	F	54	3	L1	C	Yes	Yes	Yes	D	36	2	4	No	No	0.6	− 1.0
28	M	46	25	L1	C	No	No	Yes	E	40	6	6	X1, X4	no	3	–
29	F	53	0.2	T12	C	No	No	No	E	33	6	6	No	No	3	− 2.2
30	F	54	0.3	L2	B	No	Yes	No	E	20	3	3	no	IN	2	− 1.8
31	F	28	5	L2	B	Yes	No	Yes	E	28	5	3	X2	No	6.5	–
32	M	23	0.4	T12	B	No	Yes	No	C	26	3	3	No	No	3.5	–
33	M	36	5.5	L2	C	Yes	Yes	Yes	D	22	5	5	No	CSF	4.5	–
34	M	61	0.7	T11	B	No	Yes	Yes	E	22	7	9	No	No	4.5	− 1.5
35	F	64	5	L1	C	No	No	Yes	E	25	6	6	X2, X3	No	4	− 2.0
36	M	59	0.3	T12	C	No	No	No	E	28	5	9	No	PH	5	− 1.0
37	F	63	2	T11	B	No	No	Yes	E	40	-4	0	No	IN	3.5	− 2.5
38	M	55	2	L1	C	No	Yes	Yes	E	32	5	7	X3	No	3.5	− 2.0
39	F	63	2	T11	C	No	No	Yes	D	24	1	3	X2	No	3.0	− 1.5
40	M	37	12	T12	B	Yes	No	Yes	D	32	5	5	X1, X2, X4	No	4.2	–
41	F	51	2	L1	B	No	No	Yes	E	26	2	2	No	No	3.6	− 2.0
42	F	61	2	L1	C	Yes	No	Yes	E	25	3	4	No	PH	4	− 2.0
43	F	50	10	L1	C	Yes	No	Yes	D	30	2	3	No	No	5.5	− 1.8

*M* male, *F* female, *Pre-O* pre-operation, *Post-O* within 2 weeks after operation, *Fu* final follow-up, *VAS* visual analogue scale, *CSF* cerebrospinal fluid leakage, *IN* intercostal neuralgia, *PH* pleural hematoma, *DVT* deep vein thrombosis, *AIS* American Spinal Injury Association Impairment Scale, *BMD* bone mineral density

*Type B* refers to burst facture; *Type C* refers to compression fracture; *X1* refers to the failure of screw implantation on one or both sides of the injured vertebra; *X2* refers to endplate destruction of the injured vertebra; *X3* refers to Cage falls into the injured vertebral body; *X4* refers to a sagittal displacement of the vertebral body during operation

*Collapse refers to injury and collapse of the vertebral upper endplate, lower endplate, or upper and lower endplate caused by intervertebral disc herniation

^#^*Facet fusion* refers to a spontaneous fusion of unilateral or bilateral facet joints after trauma

### Surgical technique

After tracheal intubation and general anesthesia, each patient was placed in a prone position with the abdomen suspended. After conventional disinfection, four to six pairs of screws were inserted by the Wiltse approach. The screws of the injured vertebrae were screwed out temporarily and bone wax closure was performed to allow screw placement after disc and anterior longitudinal ligament release was completed.

**Fig. 1:**
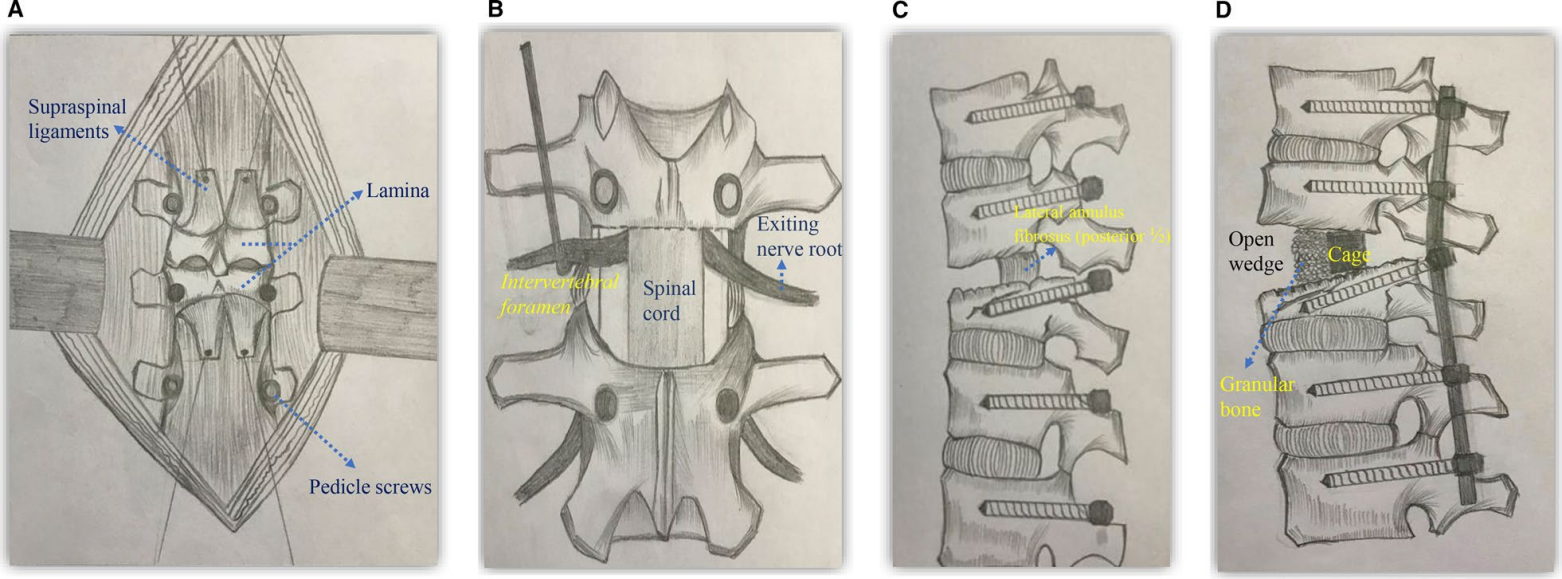
The diagram of spinal joints release technique. **A** The screws were placed through the Wiltse approach, the screws of the injured vertebra were pulled out, and the multifidus muscle was stripped from the side of the spinous process and the surface of the lamina, broken in the middle, and pulled to both sides. **B** The spinous process, lamina, and bilateral articular processes were removed and the existing nerve roots were protected. **C** After clearance of intervertebral space and disconnection of anterior longitudinal ligament and callus, release the anterior 1/2 of bilateral fibrous rings of the intervertebral disc as reduction hinge for patients who were still rigid. **D** Pre-bend the connecting rod into a physiological radian and place it alternately on both sides. There was no obvious shortening of the spine and spinal cord. Local autologous granular bone was implanted into the intervertebral space. If the posterior 1/2 of the endplate was not damaged, the local autologous granular bone was placed in the anterior 1/2 of the intervertebral space, and the Cage was placed in the posterior 1/2 of the intervertebral space

The supraspinal and interspinous ligaments and ligamentum flavum of the injured vertebrae were dissected. The new technique adopted the resection of the posterior part of the lamina (3–4 cm) of injured vertebrae and proximal vertebrae, and the spinous process and lamina were further removed to expose the dura for 3–4 cm. The bilateral articular process was removed with an ultrasonic bone knife to open the foramina (Fig. 1A-B). After taking protective measures for the exiting nerve root, the posterolateral annulus fibrosus of the intervertebral disc was found and cut horizontally with a sharp knife blade or bone knife. The posterior 3/4 nucleus pulposus and cartilage end plate in the intervertebral space were cleaned with nucleus pulposus forceps, curettes, and bone knives. The temporary connecting rod was installed on the no-operation side. At this point, we loosened the proximal nut of the temporary connecting rod, and the open-wedge anterior intervertebral disc space was seen by lateral fluoroscopy; if not, we pressed the posterior spinous process or cleaned the anterior longitudinal ligament and the callus again to achieve open-wedge anterior space. If the broken end was still stiff, lateral annulus fibrosis of the indexed disc was slowly and bluntly released from front to back by ipsilateral or bilateral alternation, preserving the posterior 1/2 of the lateral annulus fibrosus. A connecting rod of physiological curvature was used for the successful correction of kyphosis. A granular bone of 2–3 mm in size made from locally harvested bone was implanted into the space through the intervertebral foramen, and it was supported by a cage of 8–12 mm in height in the posterior half of the intervertebral space (Fig. 1C-D).

In order to protect the anterior vertebral vessels, pleura, peritoneum, and diaphragm, the following points should be paid attention to when loosening the anterior longitudinal ligament and callus in the front 1 /4 of the injured intervertebral space: (1) All operations can only be performed under the periosteum, in the ligament and in the callus with blunt instruments. (2) Blunt instruments are only limited to slowly prying and pulling up, down, left and right, and separating; lateral fluoroscopy must be used every 2 mm forward of the instrument to ensure that the instrument does not enter the front of the anterior longitudinal ligament and callus. (3) After loosening, the activity of the broken end can be obviously increased during the operation, and there is a sense of falling into the anterior intervertebral space with blunt instruments.

### Outcome measures

The operative time, intraoperative blood loss, and complications were recorded. The visual analog scale (VAS), Oswestry Disability Index (ODI) [22], and AIS [23] were recorded preoperatively, two weeks after surgery, and at the final follow-up. The local kyphosis Cobb angle was measured at a Picture Archiving and Communication Systems (PACS) workstation. According to the fusion criteria proposed by Toth [24], the postoperative bone fusion was evaluated by X-ray. Grade 1 (non-fusion) refers to the obvious light transmission line around the cage on the X-ray film. Grade 2 (possible fusion) refers to some light transmission lines around the cage. Grade 3 (strong fusion) means that there is no light transmission line around the cage. Intraoperative intervertebral space release and implant fixation segments were recorded.

Initially, TC and WGJ read one batch of CTs, X rays and developed a reading protocol for evaluating bone fusion and morphological parameters. Using this protocol, the readers (TC and WGJ) read and re-read 15 CTs and X-rays performed two weeks apart, blinded to the identity of the patient in order to assess interobserver and intra-observer reliability. Results of the interobserver and intra-observer reliability tests for bone fusion (kappa statistics) were 0.84 and 0.87; and for morphological parameters (inter-class correlation-ICC), 0.92 for interobserver and 0.86 for intra-observer, demonstrating excellent reliability.

### Statistical analysis

SPSS20.0 statistical software was used for statistical analysis. The normal distribution of continuous variable parameters, such as VAS score, using the Shapiro–Wilk test. The counting data were expressed as percentages, and the measurement data were expressed as mean ± standard deviation (SD). The values of VAS, ODI, and Cobb angle of thoracolumbar kyphosis before the operation, after the operation, and at the final follow-up were compared by one-way repeated measures analysis of variance, and *P* < 0.05 was considered statistically significant.

## Results

Data from the 43 patients with rigid post-traumatic kyphosis were shown in Table 1. All patients successfully completed the SJR operation. Open-wedge anterior intervertebral disc space was performed in 31 cases (72%), and repeated release and dissection of anterior longitudinal ligament and callus were performed in 12 cases (28%). There was no lateral annulus fibrosis release in 11 cases, the anterior half release of lateral annulus fibrosis in 27 cases, and complete release in five cases. There were five cases of screw placement failure in one or two side pedicles of the injured vertebrae, three cases of screw path destruction due to excessive resection of the superior articular process of the injured vertebrae, and two cases of screw loosening due to mismatching of the pre-bending curve and the kyphotic curve during the reduction process after screw placement. Six patients had an end plate injury caused by intraoperative clearance of the intervertebral space. Sagittal displacement occurred in four cases at the released segment due to the complete release of bilateral lateral annulus fibrosus, which was corrected by adjusting screw depth and rod pre-bending radian. Autologous granular bone + Cage was implanted in 32 cases, and autologous granular bone was implanted in 11 cases. The average operation time was 224 ± 31 min (160–260 min), and intraoperative blood loss was 450 ± 225 mL (340–700 mL). No dural rupture and no obvious intervertebral bleeding were found during the operation, and there was no thoracoabdominal aorta or inferior vena cava injury. Latent cerebrospinal fluid leakage occurred in five cases, which was cured by drainage tube extraction nine days after the operation. There were 2 cases with a small amount of pleural effusion after the operation (case 36 and case 42), which were spontaneously absorbed without treatment. Radiculopathy at the facet resection level occurred in five patients after surgery (unilateral in three cases and bilateral in two cases), and symptoms were relieved by oral mecobalamin. One patient developed deep vein thrombosis of the left lower limb 11 days after surgery, which was cured by vascular surgery. No incisional infection or neurological deterioration was discovered.

All of the patients were followed up for an average of 26 ± 8.5 months (6–37 months). The preoperative VAS score for low-back pain was 5.5 ± 1.1, which decreased to 1.1 ± 0.9 two weeks after the operation (*P* < 0.05), and 0.9 ± 0.9 at the final follow-up (*P* < 0.05). The ODI was 58.7 ± 10.6 before the operation, which decreased to 20.6 ± 5.7 two weeks after the operation (*P* < 0.05) and 11.5 ± 5.4 at the final follow-up (*P* < 0.05). Seventeen patients had an incomplete spinal cord injury before surgery, and 14 patients recovered to grade E of AIS at the final follow-up (Table 2).

**Table 2:** Changes of AIS grade in patients with RPTK at each time point

	A	B	C	D	E
Pre-op (N = 43)	0	0	3	13	26
2 weeks Post-op (N = 43)			1	4	38
Final FU (N = 37)	0	0	0	3	34

*RPTK* rigid posttraumatic thoracolumbar kyphosis, *Pre-op* pre-operation, *Post-op* post-operation, *FU* follow-up

**Fig. 2:**
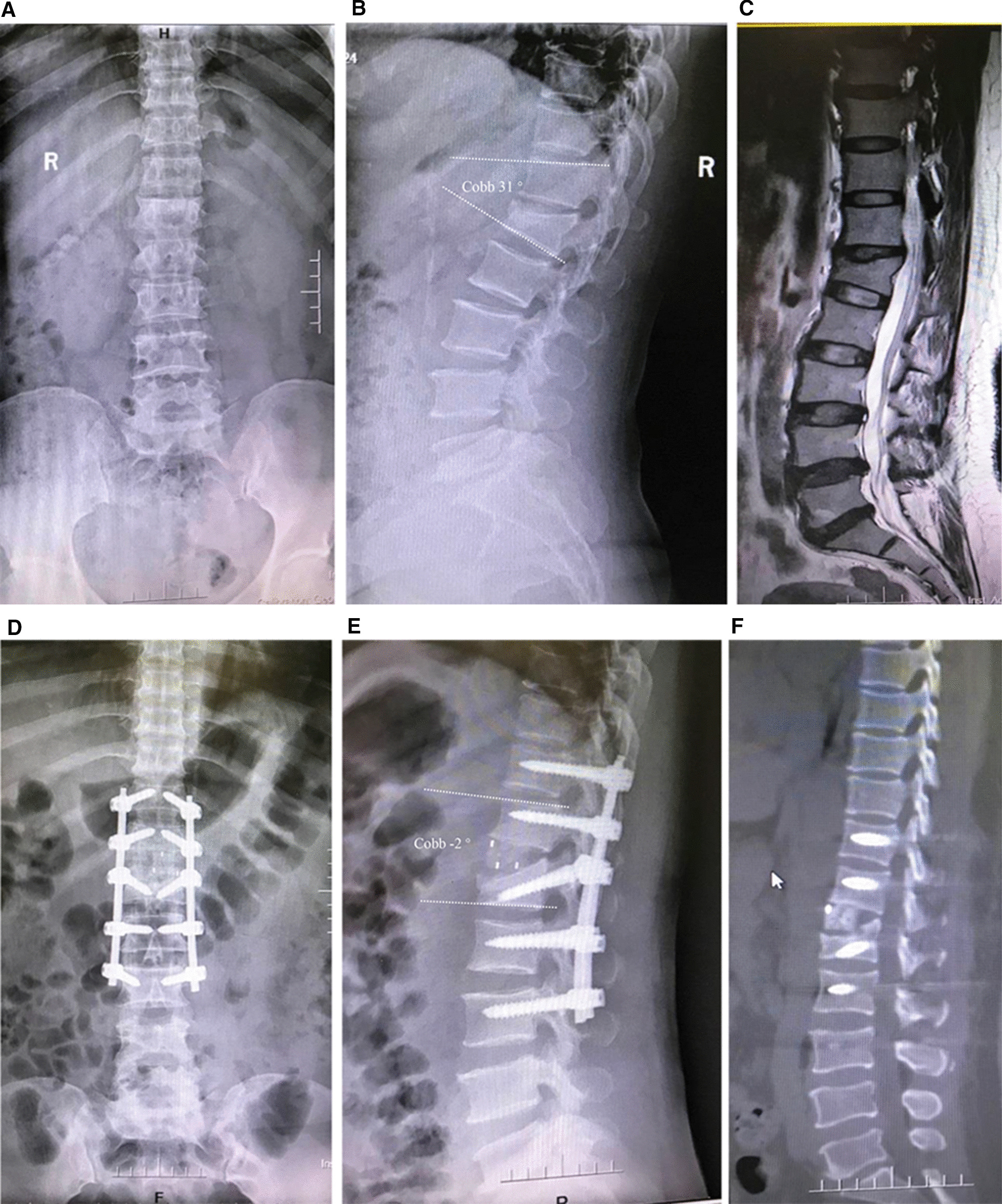
A 49 years-old female was diagnosed with posttraumatic thoracolumbar kyphosis. **A, B** The X-ray indicated that the L1 vertebra suffered from an old burst fracture with a local cobb angle of 22°kyphosis. **C** MRI showed compression of the dural sac and anterior displacement of the spinal conus. **D, E** X-ray showed that the opening of the anterior intervertebral space was good and the recovery of the spinal sequence was satisfactory two weeks after the operation. **F** CT showed that the spine sequence was good and the fixation was firm at the final follow-up

**Fig. 3:**
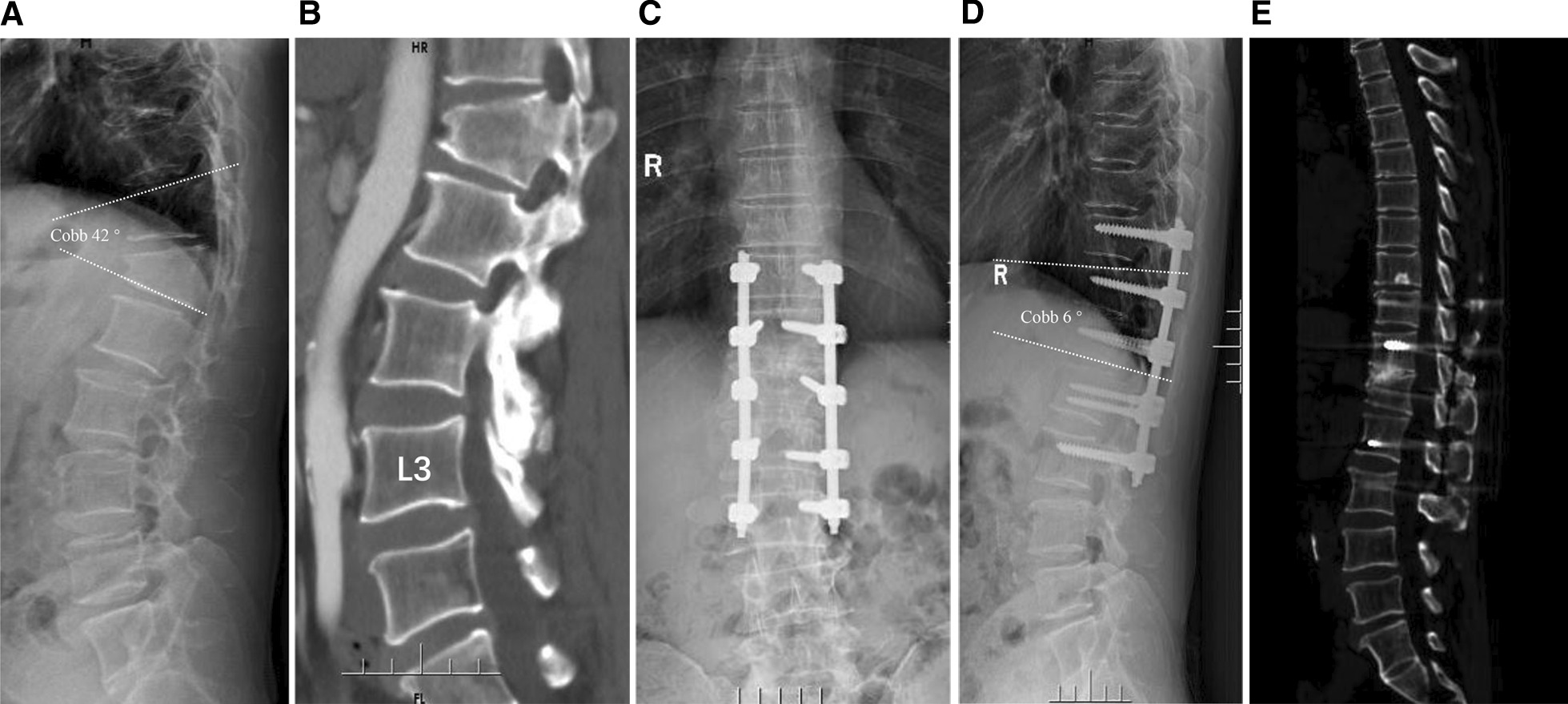
A 46 years-old male, who was diagnosed with posttraumatic thoracolumbar kyphosis, suffered from low back pain after trauma for 25 years. **A** The X-ray showed an L1 compression fracture with a severe wedge-shaped upper endplate, and the Cobb angle of thoracolumbar kyphosis was 36 degrees. **B** Angiography indicated that there was a certain gap between the anterior edge of the vertebral body and the abdominal aorta. **C–E** Three years after the operation, the spinal sequence remained satisfactory with a cobb angle of 6 degrees. CT showed T12-L1 intervertebral space bone graft fusion

Two weeks after the operation, the thoracolumbar kyphosis well recovered. However, the intervertebral graft was trapped in the injured vertebrae in five patients. No intervertebral dislocation was observed and the fixation was firm. At the final follow-up, radiographs and CT scans showed that all of the patients had bony healing (grade 2 or 3 in Toth criteria [24]) in the vertebral space and maintained good spinal alignment (Fig. 2 and Fig. 3). In this group, the average Cobb angle, which was 27.7° ± 7.8° (18°–49°) before surgery, decreased to 3.6° ± 3.5° (− 4°–11°) two weeks after surgery (*P* < 0.05), and to 5.4° ± 3.0° at the final follow-up (*P* < 0.05), with 87% correction. The Cobb angle was slightly lost at the final follow-up compared with that at two weeks after surgery (*P* < 0.05) (Table 3).

**Table 3:** Cobb angle, VAS score and ODI index (x ± s) of 43 patients with RPTK at each observation time point

	Cobb Angle (°)		VAS		ODI	
	Range	Mean	Range	Mean	Range	Mean
Pre-op	18 ~ 55	27.7 ± 7.8	4 ~ 8	5.5 ± 1.1	40 ~ 80	58.7 ± 10.6
2 weeks Post-op	− 4 ~ 11	3.6 ± 3.5^a^	0 ~ 3	1.1 ± 0.9^a^	5 ~ 30	20.6 ± 5.7^a^
Final FU	0 ~ 12	5.4 ± 3.0^b,c^	0 ~ 3	0.9 ± 0.9^b,c^	0 ~ 25	11.5 ± 5.4^b,c^
Statistical value	^a^F = 21.218; ^a^*P* < 0. 001		^a^F = 18.024; ^a^*P* < 0. 001		^a^F = 22.146; ^a^*P* < 0. 001	
	^b^F = 20.12; ^b^*P* < 0. 001		^b^F = 20.214; ^b^*P* < 0. 001		^b^F = 24.357; ^b^*P* < 0. 001	
	^c^F = 8.40; ^c^*P* = 0. 010		^c^F = 2.450; ^c^*P* = 0.135		^c^F = 8.957; ^c^*P* = 0. 008	

*RPTK* rigid posttraumatic thoracolumbar kyphosis, *Pre-op* pre-operation, *Post-op* post-operation, *FU* follow-up

^a^Comparison between 2 weeks after operation and pre-operation

^b^Comparison between final follow-up and pre-operation

^c^Comparison between final follow-up and 2 weeks after operation

## Discussion

We showed that the SJR technique can clean the intervertebral space issue of the injury segment, release the anterior longitudinal ligament, bilateral lateral fibrous rings, callus, and correct kyphosis through the anterior opening of the intervertebral space, achieving satisfactory clinical efficacy and reducing the surgical trauma and the risk of spinal cord injury.

In the recent 20 years, RPTK has achieved good clinical efficacy through PSO or modified PSO technology [5–13], but there are some complications such as a large amount of osteotomy, severe trauma, excessive blood loss, severe spinal cord shortening, and spinal cord injury. It has been reported that the classical transpedicular osteotomy technique or modified pedicle osteotomy technique removes half of the pedicle and half of the vertebral body combined with the injured disc, which can lead to intraoperative blood loss of up to 1500–2200 mL [5–9]. Hua et al. reported that the intraoperative blood loss of patients with RPTK treated by transforaminal wedge osteotomy could also reach 1220 mL [19]. The possible causes of intraoperative bleeding during PSO include the following: (1) cutting off the bilateral transverse processes of the injured vertebrae for stripping the outer edge of the pedicle and the vertebral periosteum; (2) injuring intercostal arteries or vertebral arteries directly from the thoracic or abdominal aorta; (3) cancellous bone hemorrhage caused by vertebral osteotomy; (4) injury of basivertebral vein foramen during posterior vertebral wall resection; (5) diffuse oozing caused by extensive exposure of cancellous bone of the vertebral body. The possible causes of spinal cord injury may be as follows: (1) improper and extensive excision of posterior appendix structures of the spine, such as spinous process, lamina, and articular process; (2) adhesion between the posterior wall of vertebrae and dura; (3) spinal cord injury caused by abnormal intervertebral movement during reduction; (4) excessive shortening of the spinal cord after kyphosis correction. In recent years, although some scholars have achieved good results by using lateral and posterior approaches for lumbar scoliosis and kyphosis to release intervertebral space and anterior longitudinal ligament [14, 16–18], there have been few clinical observations on the treatment of patients with RPTK.

However, we proposed a new technique to treat patients with RPTK by SJR. The new technique adopted the resection of the posterior part of the lamina (3–4 cm) of injured vertebrae and proximal vertebrae, and spinal process, bilateral joints of the injured vertebrae (Schwab II). While protecting the travel nerve root, resection of the intervertebral disc and blunt dissociation of the anterior longitudinal ligament and prevertebral scar callus were achieved, and most of the cortical bone under the end plate was retained. The mechanical stress point of the released intervertebral space is located on the posterior and lateral annulus fibrosus and/or the connecting rod. When the anterior opening and posterior wall of the intervertebral space are slightly closed by controlling the spinous process. This technique achieved circular release and decompression around the spinal cord, avoided spinal cord shortening [20, 21], and did not require extensive resection of the bilateral transverse process, pedicle, vertebrae, and posterior wall of injured vertebrae. In most patients, the posterior wall and posterior 1/2 of the lateral fibrous ring of the injured vertebral body can be retained. In addition, the biomechanical behavior of the fractured vertebrae was improved with routine screw placement. An 87% kyphotic correction rate at the final follow-up was obtained in our study, and the average blood loss during surgery was 450 mL. None of the patients had severe hemopneumothorax spinal cord injury, and postoperative intervertebral foraminal nerve root squeezing injury was healed by symptomatic treatment for 3–6 months. After more than 2.6 years of follow-up, satisfactory clinical results were obtained.

Although SJR is an effective treatment for RPTK, with good clinical and imaging results, there are still some complications. The following points should be noted during the operation: (1) the foramina should be fully exposed in the safe triangle area (the triangle formed by the dural sac, nerve roots, and the outer edge of the foramina). The outlet nerve roots should be protected to avoid pulling and cutting them. At the same time, when excising the base of the superior articular process of the injured vertebra, attention should be paid to protecting the screw path of the injured vertebra from damage. (2) Some patients could not find the space due to intervertebral stenosis and the ossification of the posterior annulus fibrosus. It is important to observe the position and tilt direction of the intervertebral space through fluoroscopy before cutting the posterior lateral annulus fibrosus with a sharp knife or bone knife. (3) The disc that has collapsed into the vertebra need not be removed, but the hardened end plate of the injured vertebrae should be protected to avoid bleeding caused by entering the vertebral body and reduce the supporting function of the intervertebral fusion device. (4) The resection and release of the anterior 1/4 of the intervertebral space should be slowly advanced, cured up and down, and pried off. C-arm fluoroscopy should be repeatedly used to clarify the position and depth of the instrument so as to avoid damage to the prevertebral vessel and diaphragm. Therefore, preoperative multidisciplinary consultation (including departments of thoracic surgery and vascular surgery) is necessary. (5) After the anterior release of the intervertebral space, a pre-bent rod with physiological curves was adopted correctly so as to prevent the dislocation and rotation of the unstable vertebra and screw removal from the injured vertebrae. (6) Bilateral lateral annulus fibrotomy should be determined based on the stability between the fractured ends of the spine and correction of the deformity after clearance of the intervertebral space and anterior longitudinal ligament and callus resection. Some patients do not need the release of the lateral annulus. In this study, bilateral lateral annulus fibrotomy at anterior 1/2 was performed in 27 cases. There was no sagittal displacement. However, four of five patients with completed release had a sagittal displacement. Thus, for patients with long injury course and severe kyphosis requiring bilateral lateral annulus fibrotomy, it is recommended to partially release the lateral annulus fibrosus from the front to the back and preserve the posterior half of bilateral annulus fibrosus as far as possible. (7) Intervertebral braces should be measured and placed after unilaterally fixed rods are placed. If the intervertebral space is too small, the end plate is damaged, or the end plate is collapsed without support function, it is necessary to place only local granular bone packing. During the placement of intervertebral braces, the front half of the end plate of the intervertebral space is mostly injured by the manipulation of instruments, and the intervertebral support fusion material placed behind the half of the intervertebral space could have stronger support. (8) Extensive calcification of the abdominal aorta may be contraindicated.

## Data Availability

Data will be available upon request to the first author, WQ.

## References

[1] Munting E (2010). Surgical treatment of post-traumatic kyphosis in the thoracolumbar spine: indications and technical aspects. Eur Spine J.

[2] Wang Q, Xiu P, Zhong DJ (2012). Simultaneous posterior and anterior approaches with posterior vertebral wall preserved for rigid post-traumatic kyphosis in thoracolumbar Spine. Spine.

[3] Suk SI, Kim JH, Lee SM (2003). Anterior-posterior surgery versus posterior closing wedge osteotomy in posttraumatic kyphosis with neurologic compromised osteoporotic fracture. Spine (Phila Pa 1976).

[4] Benli IT, Kaya A, Uruc V (2007). Minimum 5-year follow-up surgical results of post-traumatic thoracic and lumbar kyphosis treated with anterior instrumentation: comparison of anterior plate and dual rod systems. Spine.

[5] Schwab F, Blondel B, Chay E (2014). The comprehensive anatomical spinal osteotomy classification. Neurosurgery.

[6] Gao R, Wu J, Yuan W (2015). Modified partial pedicle subtraction osteotomy for the correction of post-traumatic thoracolumbar kyphosis. Spine J.

[7] Hu W, Wang B, Run H (2016). Pedicle subtraction osteotomy and disc resection with cage placement in post-traumatic thoracolumbar kyphosis, a retrospective study. J Orthop Surg Res.

[8] Liu FY, Gu ZF, Zhao ZQ, Ren L, Wang LM, Yu JH, Hou SB, Ding WY, Sun XZ (2020). Modified grade 4 osteotomy for the correction of posttraumatic thoracolumbar kyphosis: a retrospective study of 42 patients. Medicine.

[9] Xi YM, Pan M, Wang ZJ (2013). Correction of post-traumatic thoracolumbar kyphosis using pedicle subtraction osteotomy. Eur J Orthop Surg Traumatol.

[10] Malcolm BW, Bradford DS, Winter RB (1981). Post-traumatic kyphosis. A review of forty-eight surgically treated patients. J Bone Joint Surg.

[11] Gertzbein SD, Harris MB (1992). Wedge osteotomy for the correction of post-traumatic kyphosis: a new technique and report of three cases. Spine.

[12] Lazennec JY, Neves N, Rousseau MA (2006). Wedge osteotomy for treating post-traumatic kyphosis at thoracolumbar and lumbar levels. J Spine Disord Tech.

[13] Suk S, Kim J, Lee S (2003). Anterior-posterior surgery versus posterior closing wedge osteotomy in post-traumatic kyphosis with neurologic compromised osteoporotic fracture. Spine.

[14] Mundis GM (2017). Anterior column realignment has similar results to pedicle subtraction osteotomy in treating adults with sagittal plane deformity. World Neurosurg..

[15] Bohl MA, Hlubek RJ, Xu DS (2020). Posterior open-wedge anterior longitudinal ligament release (POWAR): cadaveric technique analysis. Science.

[16] Park P, Wang MY, Lafage V (2015). Comparison of two minimally invasive surgery strategies to treat adult spinal deformity. J Neurosurg Spine.

[17] Saigal R, Mundis GM, Uribe J (2016). Anterior column realignment (ACR) in adult sagittal deformity correction: technique and review of the literature. Spine (Phila Pa 1976).

[18] Sweet FA, Sweet A (2015). Transforaminal anterior release for the treatment of fixed sagittal imbalance and segmental kyphosis, minimum 2-year follow-up study. Spine Deform.

[19] Hua Q, Zhao HY, Chen YJ (2016). treatment of transforaminal wedge osteotomy for correction of the thoracolumbar kyphosis. Chin J Orthop.

[20] Kawahara N, Tomita K, Kobayashi T (2005). Influence of acute shortening on the spinal cord: an experimental study. Spine (Phila Pa 1976).

[21] Alemdaroglu KB, Atlihan D, Cimen O (2007). Morphometric effects of acute shortening of the spine: the kinking and the sliding of the cord, response of the spinal nerves. Eur Spine J.

[22] Fairbank JC, Pynsent P (2000). The oswestry disability index. Spine.

[23] Rupp R (2021). International standards for neurological classification of spinal cord injury: revised 2019. Top Spinal Cord Injury Rehabili.

[24] Toth JM, Wang M, Estes BT (2006). Polyethe retherket one as a biomaterial for spinal applications. Biomaterials.

